# Aldosterone-producing adenoma and other surgically correctable forms of primary aldosteronism

**DOI:** 10.1186/1750-1172-5-9

**Published:** 2010-05-19

**Authors:** Laurence Amar, Pierre-François Plouin, Olivier Steichen

**Affiliations:** 1 Université Paris Descartes; Assistance Publique-Hôpitaux de Paris, Hôpital Européen Georges Pompidou, Hypertension and Clinical Research units, 20 rue Leblanc, 75908 Paris cedex 15, France; 2 Université Pierre et Marie Curie Paris-6; Assistance Publique-Hôpitaux de Paris, Hôpital Tenon, Service de Médecine Interne, Paris, France

## Abstract

Surgically correctable forms of primary aldosteronism are characterized by unilateral aldosterone hypersecretion and renin suppression, associated with varying degrees of hypertension and hypokalemia. Unilateral aldosterone hypersecretion is caused by an aldosterone-producing adenoma (also known as Conn's adenoma and aldosteronoma), primary unilateral adrenal hyperplasia and rare cases of aldosterone-producing adrenocortical carcinoma. In these forms, unilateral adrenalectomy can cure aldosterone excess and hypokalemia, but not necessarily hypertension. The prevalence of primary aldosteronism in the general population is not known. Its prevalence in referred hypertensive populations is estimated to be between 6 and 13%, of which 1.5 to 5% have an aldosterone-producing adenoma or primary unilateral adrenal hyperplasia. Taking into account referral biases, the prevalence of surgically correctable primary aldosteronism is probably less than 1.5% in the hypertensive population and less than 0.3% in the general adult population. Surgically correctable primary aldosteronism is sought in patients with hypokalemic, severe or resistant forms of hypertension. Recent recommendations suggest screening for primary aldosteronism using the aldosterone to renin ratio. Patients with a raised ratio then undergo confirmatory suppression tests. The differential diagnosis of hypokalemic hypertension with low renin includes mineralocorticoid excess, with the mineralocorticoid being cortisol or 11-deoxycorticosterone, apparent mineralocorticoid excess, pseudo-hypermineralocorticoidism in Liddle syndrome or exposure to glycyrrhizic acid. Once the diagnosis is confirmed, adrenal computed tomography is performed for all patients. If surgery is considered, taking into consideration the clinical context and the desire of the patient, adrenal vein sampling is performed to detect whether or not aldosterone hypersecretion is unilateral. Laparoscopic surgery for unilateral aldosterone hypersecretion is associated with a morbidity of about 8%, with most complications being minor. It generally results in the normalization of aldosterone secretion and kalemia, and in a large decrease in blood pressure, but normotension without treatment is only achieved in half of all cases. Normotension following adrenalectomy is more frequent in young patients with recent hypertension than in patients with long-standing hypertension or a family history of hypertension.

## 

This review deals with the prevalence, presentation, diagnosis and management of surgically correctable forms of primary aldosteronism (PA).

## Disease name and synonyms

PA is also called primary hyperaldosteronism. Surgically correctable forms of the condition are characterized by unilateral aldosterone hypersecretion. They include aldosterone-producing adenoma, also termed Conn's adenoma or aldosteronoma; aldosterone-producing carcinoma, a very rare condition; and primary unilateral adrenal hyperplasia, a condition with a unilateral aldosterone hypersecretion documented by adrenal vein sampling (AVS) but without a typical adenoma. In contrast, idiopathic adrenal hyperplasia and familial hyperaldosteronisms type 1 and 2, in which aldosterone hypersecretion is bilateral, are not surgically correctable. The subtypes of PA are presented in Table 1.

**Table 1:** Primary aldosteronism subtypes

Surgically correctable subtypes:

Aldosterone-producing adenoma (alias Conn's adenoma, aldosteronoma), including:
Renin- or angiotensin-unresponsive adenoma
Renin- or angiotensin-responsive adenoma
Primary unilateral adrenal hyperplasia
Adrenocortical carcinoma with aldosterone hypersecretion

Non surgically correctable subtypes:
Idiopathic adrenal hyperplasia
Familial diseases:
Familial hyperaldosteronism type I (alias glucocorticoid-remediable aldosteronism), OMIM # 103900
Familial hyperaldosteronism type II, OMIM # 605635

## Definition

Hyperaldosteronism is a condition caused by the overproduction of aldosterone, and is characterized by sodium retention and potassium excretion with resultant hypertension and hypokalemia. The condition was first described by J Conn [1], who further distinguished primary and secondary hyperaldosteronism on the basis of plasma renin levels, PA being characterized by renin suppression [2]. In a recent clinical practice guideline, PA was defined as "a group of disorders in which aldosterone production is inappropriately high, relatively autonomous from the renin-angiotensin system, and non-suppressible by sodium loading" [3].

## Epidemiology

### Prevalence

**Figure 1:**
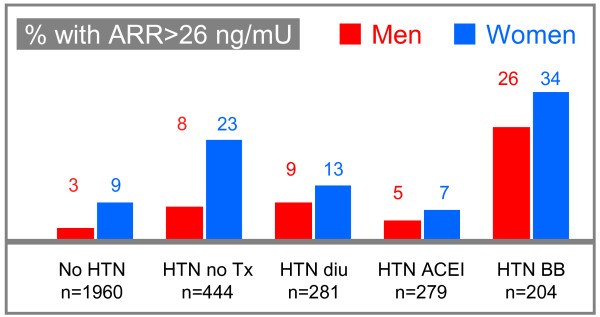
**Prevalence of subjects with an elevated ARR**. This figure, derived from the article of Newton-Cheh et al [4], shows the prevalence of an elevated aldosterone to renin ratio (ARR) among subjects with or without hypertension (HTN) in relation to various antihypertensive treatments (Tx): Diu, diuretics; ACEI, angiotensin-converting enzyme inhibitors; BB, beta-blockers.

The prevalence of PA and its various surgically correctable subtypes in adults is not known. The prevalence of a raised aldosterone to renin ratio in the general population [4] (Figure 1) and in hypertensive patients referred to specialized centers [5-9] is high (Table 2), but a raised ratio is not sufficient for diagnosing PA (see [3] and the Diagnosis section below). In referral samples involving more than 1,000 hypertensive patients [5-10], the prevalence of a raised aldosterone to renin ratio ranged from 6.4 to 22.8%: 5.9 to 11.3% of the patients were confirmed with PA, and 1.5 to 4.8% had an aldosterone-producing adenoma. These figures have probably been overestimated due to referral biases. Assuming (a) a prevalence of hypertension of 20% in adult subjects aged 60 [11] or less in whom adrenalectomy would be considered (see Prediction of blood-pressure outcome below), (b) a conservative two-fold estimate of the over-representation of PA in hypertensive patients referred to specialized clinics, and (c) a 3% prevalence of aldosterone-producing adenomas in referred hypertensives, the prevalence of surgically correctable PA in those aged between 18 and 60 years is less than 1.5% in the hypertensive population and less than 0.3% in this age group in the general population. In addition to the low prevalence of surgically correctable PA, some patients do not undergo surgery and only one in two operated patients becomes normotensive without medication following an adrenalectomy (see Management and Prognosis below).

**Table 2:** Prevalence of a raised aldosterone to renin ratio and of aldosterone-producing adenomas in referral samples or samples from a large community

Source and first author	ARR threshold, ng/dL per ng/mL.h^-1^	Subjects tested, n	Raised ARR, %	Confirmed PA, %	Proven adenoma, %
Community sample of hypertensive and non-hypertensive subjects					

Newton-Cheh C, 2007[4]	21*				

Men		1574	7	NR	NR

Women		1752	13	NR	NR

Referral samples of >1000 hypertensive patients					

Nishikawa T, 2000[5]	20	1020	6.4	NR	4.2

Rossi E, 2002[10]	35	1046	12.8	6.3	1.5

Rossi GP, 2006[7]	40	1125	20.4	11.2	4.8

Fogari R, 2007[8]	25	3000	22.8	5.9	1.8

Douma S, 2008[9]	30	1616	20.9	11.3	NR

ARR: aldosterone to renin ratio. PA: primary aldosteronism

Screening tests were considered positive on the basis of a high ARR alone [5,7-9], a composite criterion[7], or the combination of a high ARR and a plasma aldosterone concentration of 416 pmol/l (15.0 ng/dL) or more[9]. PA was generally confirmed by a sodium suppression test.

* Direct renin concentration (mU/l) determined by an automated method was converted to plasma renin activity using a conversion factor of 8.2 (see Funder JW et al, 2008[3])

Adrenalectomy for Conn's adenoma has been reported in childhood [12]. The prevalence of surgically correctable PA in children and adolescents is not known but is probably very low.

### Incidence

Between 1977 and 1981, the incidence rate of aldosterone-producing adenomas for which the patient underwent surgery was estimated at 0.8 per million individuals per year in Denmark [13]. This figure is a low estimate, as it was obtained at a time when the aldosterone to renin ratio was not in use and computed tomography was not widely available. A national epidemiological survey in Japan estimated that 1,450 patients had been diagnosed with PA of any form in 1997 [14]. In 1997, there were about 70 million Japanese adults aged 60 or less; thus, the incidence rate of PA could be estimated to be 2 cases per 100,000 individuals per year in this age group.

## Clinical description

Patients with PA present with various degrees of hypertension and/or hypokalemia.

Surgically correctable PA is usually diagnosed in the fourth or fifth decade. Mean age at PA diagnosis across 9 large series ranged between 45 and 55 years, with an overall average of 50 years [15-24]. Hypertension was generally detected 5 to 10 years before PA was diagnosed, indicating a significant delay between the onset of PA and its diagnosis. Grade III hypertension - with blood pressure (BP) levels of 180/110 mmHg or more - or resistant hypertension - with BP levels of 140/90 mmHg or more on triple antihypertensive treatment - are reported more frequently in patients with PA than in those with essential hypertension [7,25]. The higher frequency of severe hypertension in patients with PA than in those with essential hypertension may be the consequence of an exploration bias, as current recommendations suggest screening for secondary causes, including PA, in patients with severe or difficult-to-treat hypertension [26]. Left ventricular hypertrophy, microalbuminuria, and acute cardiovascular events are probably more frequent in patients with PA than in patients with essential hypertension and similar levels of BP (see Treatment objectives below).

Hypokalemia, usually defined as serum kalemia ≤ 3.5 mmol/l, is only present in a minority of patients with PA [5-9]. However, the frequency of hypokalemia is related to whether PA can be surgically cured. A study with a large series of patients reported that hypokalemia was present in 7%, 17% and 48% of patients with essential hypertension, idiopathic PA, and aldosterone-producing adenoma, respectively [7]. Hypokalemia may be symptomatic and present as muscular weakness, cramps, paresthesia or palpitations with or without atrial fibrillation. There are rare cases in which PA is revealed by symptomatic hypokalemia without hypertension, with or without adenoma [27].

PA may also be documented in patients presenting with an incidentally detected adenoma. In a survey of 1096 patients with an adrenal 'incidentaloma', 16 patients were found to have PA, all of whom were moderately hypertensive [28].

## Etiology

### Etiology of primary aldosteronism

The aldosterone to renin ratio is a heritable trait with a moderate degree of linkage to chromosome 11p [4]. The etiology of aldosterone-producing adenoma and primary unilateral hyperplasia is not known.

### Mechanism of hypertension in primary aldosteronism

The main effects of aldosterone are mediated by the mineralocorticoid receptor found in the cytosol of epithelial cells, particularly in the renal collecting duct. Aldosterone's major action on epithelial cells is to regulate the reabsorption of Na+, thereby also influencing the transport of water, K+, and H+ across the membrane. An electrochemical gradient permits the passage of sodium from the lumen into the epithelial cell through the amiloride-sensitive epithelial sodium channel. From there, active transport by the Na+/K+-ATPase carries the Na+ across the basolateral membrane, from the epithelial cell into the bloodstream, while simultaneously excreting K+; water follows the movement of the Na+. Aldosterone hypersecretion therefore increases exchangeable sodium, suppresses renin, increases the aldosterone to renin ratio, causes hypertension, and induces hypokalemia.

## Diagnosis of primary aldosteronism

**Figure 2:**
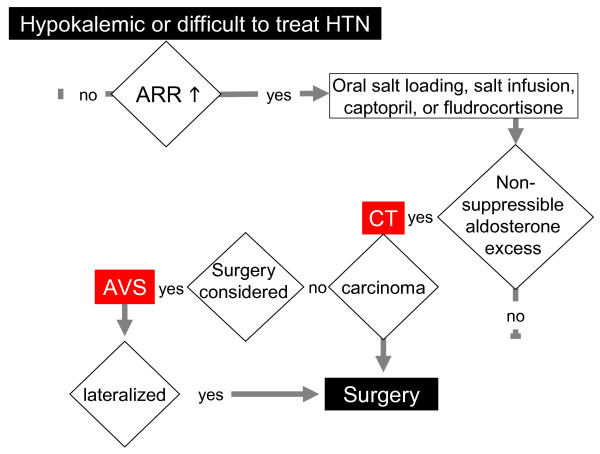
**Algorithm for screening, diagnosis and management of primary aldosteronism**. This algorithm, drawn from recent guidelines[3], suggests screening patients with difficult-to-treat or hypokalemic hypertension using the aldosterone to renin ratio (ARR). Patients with a raised ARR undergo confirmatory tests. Adrenal computed tomography (CT) is performed in patients with confirmed PA. Adrenal vein sampling (AVS) is suggested to patients who may have an adrenalectomy, to identify whether or not aldosterone hypersecretion is unilateral.

Recent guidelines suggest screening for PA patients with difficult-to-treat or hypokalemic hypertension using the aldosterone to renin ratio. Patients with a raised aldosterone to renin ratio then undergo a confirmatory test. Adrenal computed tomography is performed in patients with confirmed PA to study the morphology of the adrenal glands. AVS is then suggested for patients considering an adrenalectomy, to investigate whether or not aldosterone hypersecretion is unilateral (Figure 2) [3].

### The aldosterone to renin ratio as a screening test

Aldosterone secretion increases when standing upright and with decreasing sodium intake. It decreases with decreasing plasma potassium concentrations and increasing age. Renin levels are also increased by decreases in sodium intake and the standing position, and they also decrease with increasing age. Therefore, using the aldosterone to renin ratio decreases the intra- and inter-patient variability in renin and aldosterone levels linked to sodium intake, body position and age[29,30]. The aldosterone to renin ratio was introduced by K Hiramatsu and colleagues in 1981 as a screening tool to facilitate the diagnosis of PA among hypertensive patients [31].

Although logical and convenient, using the aldosterone to renin ratio has several limitations. First, renin levels can be determined as either plasma renin activity or active renin concentration[32], and aldosterone can be determined with iodinated or tritiated markers, with or without an extraction step. Consequently, reference values and diagnostic thresholds for renin, aldosterone and the aldosterone to renin ratio are laboratory-specific. Second, there is no agreement on the aldosterone to renin ratio cut-off value for diagnosing PA. In a systematic literature review, the aldosterone to plasma renin activity cut-off values suggested ranged from 7.2 to 100 ng/dl per ng/ml.h, corresponding to a 14-fold variation [33]. The most frequently used cut-off values for aldosterone to plasma renin activity ratio are in the range of 20 to 50 ng/dl (554 to 1,385 pmol/l) per ng/ml.h; for aldosterone to active renin concentration, these values are in the range of 2.4 to 4.9 ng/dl (66 to 136 pmol/l) per mU/L (see [3] and Table 2). Third, the aldosterone to renin ratio is positively related to age, female sex, hypertensive status, and the use of beta-blockers or hormonal replacement therapy, and is negatively related to the use of angiotensin-converting enzyme inhibitors, angiotensin-receptor blockers and diuretics[4]. The aldosterone to renin ratio may be abnormally high in patients with normal aldosterone levels and very low renin levels, specifically in some elderly patients, some patients with a high sodium intake or those taking beta-blockers, in whom renin is undetectable. Some experts have therefore suggested that renin values below an appropriate minimal value (2.5 to 5 mU/l) should not be used to calculate the aldosterone to renin ratio [7,22].

Some authors suggest screening for PA without the discontinuation of medication [34]. However, antihypertensive agents alter the aldosterone to renin ratio. For example, in the Framingham Offspring Cohort, an aldosterone to renin ratio exceeding the value suggestive of PA was present in 3.1% of normotensive men and 8.8% of normotensive women, in 7.9% and 23.1% of untreated hypertensive men and women, and was present in 31.1% of men or women on beta-blockers (Figure 1)[4]. Therefore, diuretics and antihypertensive agents should be discontinued for at least two weeks and spironolactone, eplerenone and aliskiren for at least six weeks before determining the aldosterone to renin ratio. In cases in which a complete therapeutic washout would not be safe, antihypertensive medication should be limited to non-dihydropyridine calcium channel-blockers and alpha-blockers, which interfere minimally with the measurements[35]. Hypokalemic patients are given potassium chloride for two reasons: to prevent arrhythmia and because hypokalemia inhibits aldosterone secretion, thereby increasing the risk of false negative results[36]. In addition to antihypertensive agents, drospirenone, a progestin with antimineralcorticoid activity, may interfere with laboratory screening and confirmatory testing for the diagnosis of PAL and should be withdrawn in hypertensive women investigated for aldosteronism[37].

### Diagnostic confirmation

Renin-aldosterone dissociation is a key element of all definitions of PA. A raised aldosterone to renin ratio is a sensitive but non-specific test ([2-9] and Table 2). Biochemical confirmatory tests are therefore necessary in patients with a positive aldosterone to renin ratio, to avoid costly and invasive imaging tests.

The recent clinical practice guidelines for case detection, diagnosis, and treatment of patients with PA recommends that patients with a positive aldosterone to renin ratio undergo any of four suppression tests to confirm or exclude the diagnosis of PA [3]. This implies that PA is defined as a non-suppressible aldosterone excess. This approach might lead to false negatives, as some patients diagnosed with 'angiotensin-responsive' aldosterone-producing adenoma (*i.e*. with suppressible aldosterone hypersecretion) can be cured by unilateral adrenalectomy[38,39]. However, this condition is probably rare. The four suppression tests respectively use oral sodium loading, oral fludrocortisone, oral captopril, or saline infusion to suppress aldosterone secretion (Table 3). The saline infusion test has been carefully analyzed by Rossi et al. [40]. The sensitivity and specificity of a post-infusion aldosterone cutoff value of 6.8 ng/dl (188 pmol/l) were 73 and 76%, respectively. However, included patients had very high aldosterone to renin ratios and these results may not apply to patients selected with a lower cutoff. The guideline underlines that, generally, suppression tests 'have been evaluated only retrospectively, in relatively small series of patients selected with high prior (pretest) probability of PA, commonly in comparison with other tests rather than towards a conclusive diagnosis of PA' (such as the presence of a lateralized aldosterone hypersecretion or the outcome of adrenalectomy). Thus, further work is needed to improve confirmatory testing in patients with a high aldosterone to renin ratio.

**Table 3:** Suppression tests intended to confirm PA

Test	Procedure	Assays	Threshold
Oral sodium loading test	Increase sodium intake to >200 mmol/d for 3 d, provide ClK to keep plasma K+	Urinary aldosterone determined from the morning of d 3 to the morning of d 4	PA unlikely if urinary aldosterone <10 μg/24 h PA likely if urinary aldosterone >12 μg/24 h

Saline infusion test	Patient in recumbent position for at least 1 h, 2 liters of 0.9% saline iv over 4 h, starting at 0800-0930 h	Kalemia, aldosterone and cortisol at the beginning and the end of the test	PA unlikely if plasma aldosterone <5 ng/dl PA likely if plasma aldosterone >10 ng/dl

Fludrocortisone suppression test	0.1 mg oral fludrocortisone every 6 h for 4 d. Provide slow-release KCl to keep plasma K+ and slow release NaCl to maintain urinary sodium excretion >3 mmol/kg body weight	Kalemia 4 times a day during the 4 days. On day 4 determine plasma cortisol, aldosterone and PRA in seated posture at 1000 h	PA likely if upright plasma aldosterone >6 ng/dl on day 4 at 1000 h

Captopril challenge test	25-50 mg captopril orally after sitting for at least 1 hour. Patient in seated position for 1 or 2 hours	Plasma aldosterone, PRA and cortisol before and 1 or 2 hours after captopril	PA likely if plasma aldosterone is not suppressed by captopril

Adapted from [3]. Abbreviations: PA: primary aldosteronism; PRA: plasma renin activity

**Figure 3:**
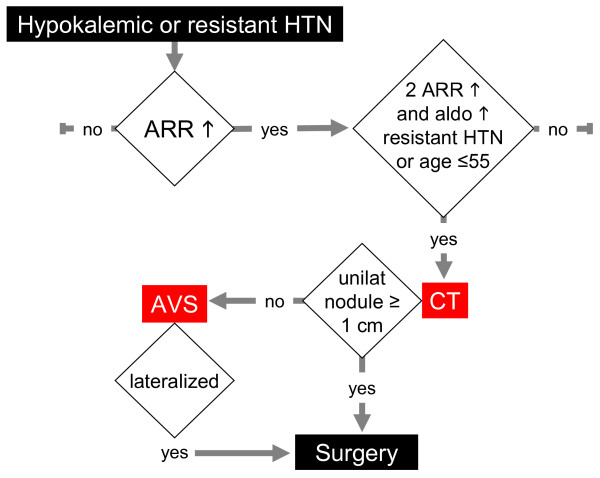
**Screening, diagnosis and management of primary aldosteronism: an alternative algorithm**. In this approach[22], PA is identified by the presence of absolute aldosterone hypersecretion, i.e. the combination of a high aldosterone to renin ratio plus a high level of plasma or urinary aldosterone documented from two separate hormonal measurements. HTN: hypertension. ARR: aldosterone to renin ratio. CT: computed tomography. AVS: adrenal vein sampling.

An alternative approach for confirming PA is to determine whether the patient has absolute aldosterone hypersecretion, which is present if there is a combination of a high aldosterone to renin ratio and a high level of plasma or urinary aldosterone [22,41,42]. In one study, 68 out of 347 hypertensives (16.6%) had a raised aldosterone to renin ratio (>25 ng/dl per ng/ml/h); only 26 (7.5%) also had a raised serum aldosterone concentration (>8 ng/dl), of whom only 11 (3.2%) also had a high urine aldosterone excretion rate (>17 μg/24 h)[41]. One study, taking into account the variability in repeated aldosterone and aldosterone to renin ratio determinations[43], considered PA to be present if two separate measurements showed a high aldosterone to renin ratio (>63 pmol/mU) plus high plasma (>500 pmol/l while lying or >550 pmol/l while standing) or urinary (>63 nmol/day) aldosterone levels[22] (Figure 3). It is however being debated whether this approach leads to false negatives. In one report, 4 of 20 patients with unilateral aldosterone hypersecretion at AVS had normal baseline serum aldosterone concentrations[44]. The precise frequency of this hormonal profile and the outcome of the adrenalectomy in those concerned are unknown.

## Diagnosis of surgically correctable primary aldosteronism

As mentioned above, cases with unilateral aldosterone hypersecretion, including the classic aldosterone-producing Conn's adenoma, are surgically correctable forms of PA. Unilateral aldosterone hypersecretion should be confirmed by AVS in most or perhaps all cases (see [3] and Figure 2). Unfortunately, AVS is not widely available, and is an invasive test exposing patients to potential complications.

### Imaging tests

**Figure 4:**
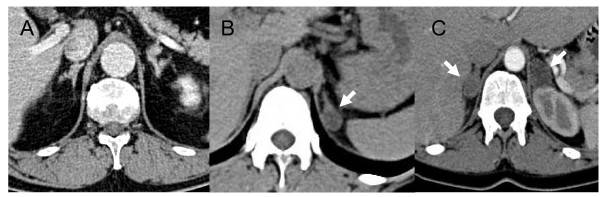
**Computed tomography of the adrenals in three patients with primary aldosteronism**. Note the moderate hypertrophy of both adrenal glands in A, the presence of a left hypodense nodule (arrow) in B with a thin right adrenal gland, and the presence of bilateral nodules (arrows) in C. All three aspects may coexist with uni- or bilateral aldosterone hypersecretion, which must be confirmed using adrenal vein sampling. In the patient with the computed tomography shown in C, the aldosterone to cortisol ratio was 1.6 in the inferior vena cava, 0.24 in the left adrenal vein and 42 in the right adrenal vein, indicating the presence of a right aldosterone hypersecretion associated with non-secreting left adenoma.

Computed tomography is the most widely used imaging test, but magnetic resonance imaging performs similarly[45]. If thin-slice computed tomography shows a single hypodense nodule, with the rest of the ipsilateral and contralateral glands appearing smooth and non-enlarged, the patient is diagnosed with isolated adrenal adenoma (Figure 4). The adrenal body and limbs are generally thicker on the left side than on the right side[46], making it difficult to diagnose isolated right adrenal adenoma in some cases. Most aldosterone-producing adenomas are less than 20 mm in diameter [22,47].

Non-secreting adenomas are present in about 2% of adult non-cancer patients and their prevalence increases with age [48]. The presence of an adenoma in patients with PA suggests the presence of an aldosterone-producing adenoma, but it cannot exclude the combination of a non-secreting adenoma and idiopathic PA[3,44]. The proportion of patients with a unilateral nodule on their computed tomography scan or magnetic resonance imaging, but a bilateral or contralateral secretion documented by AVS, was about 20% in a meta-analysis of 38 studies[49]. Nonetheless, a non-secreting adenoma is unlikely in young patients with PA and the presence of an isolated characteristic adrenal adenoma > 1 cm in PA patients aged less than 40 [6,50] or less than 55[22] is considered by some experts as an acceptable surrogate for diagnosing unilateral aldosterone hypersecretion.

Recent guidelines recommend computed tomography in all patients with confirmed PA to detect an adrenal carcinoma, even if an adrenalectomy is not otherwise considered[3]. Adrenal carcinomas are very rare, with an annual incidence estimated at 1-2 per million population, and present as isolated PA in less than 5% of cases [51]. Adrenal carcinomas presenting as PA are usually larger than 40 mm in diameter.

### Adrenal vein sampling

AVS involves determining aldosterone and cortisol levels in the inferior vena cava and in the two adrenal veins. Considering the variability in ACTH secretion and the acute control of aldosterone secretion by ACTH, some experts advocate AVS during exogenous ACTH infusion[50]. Other experts suggest AVS in the early morning, at the time of the spontaneous peak of ACTH secretion[3]. A comparative study reported that exogenous ACTH infusion does not improve the detection of unilateral aldosterone hypersecretion if the two adrenal veins are catheterized simultaneously[52].

Catheter insertion is considered successful if cortisol concentrations are two to three times higher in the adrenal veins than in the inferior vena cava or a peripheral vein. Aldosterone concentrations in both adrenal veins are then divided by the corresponding cortisol concentrations. Aldosterone secretion is considered to be lateralized if the aldosterone to cortisol ratio is two to five times higher on the dominant side than on the non-dominant side (Table 4).

**Table 4:** Proposed thresholds for interpreting results from adrenal vein sampling

	Adrenal to IVC cortisol ratio	Dominant to non-dominant A/C ratio	Non-dominant to IVC A/C ratio
**Adrenal venous sampling without ACTH stimulation**			

Rossi, 2008[21]	>1.1	>2	Not used

Stowasser, 2004[44]	>3	>2*	<1

Zarnegar, 2008[23]	>1	>4 or 5	Not used

Letavernier,2008[22]	>2	>5	Not used

Mulatero, 2008[80]	>2	>4	<1

**Adrenal venous sampling with ACTH stimulation**			

Espiner, 2003[81]	>2	>4	<1

Murashima, 2008[82]	>10	>4	Not used

Young, 2004[50]	>5	>4	Not used

Auchus, 2009[83]	>3	>4	Not used

Interpreting adrenal vein sampling results involves accounting for the ratio of cortisol concentrations between the adrenal gland veins and the inferior vena cava (IVC) or a peripheral vein, to confirm that adrenal gland veins have been effectively cannulated. The lateralizing ratio, i.e. the ratio between the aldosterone to cortisol (A/C) ratios determined in the dominant (D) and non-dominant (ND) sides, is also considered (see text).

*Dominant to IVC A/C ratio

AVS is a relatively complex procedure, with rates of failure (documented by a cortisol concentration in the cannulated vein(s) below two times higher than those in the inferior vena cava) between 3 [53] and 22% [54]. It is invasive, and carries a risk of complications of between 0.2 [53] and 5% [55]; complications mainly include adrenal hematomas, groin hematomas and dissection of adrenal veins [56]. However, AVS is superior to image-based techniques for therapeutic decisions, because the objective of surgery is to suppress unilateral hypersecretion, not a unilateral nodule. As mentioned above, a unilateral adenoma is compatible with an incidentaloma associated with idiopathic PA, particularly in elderly patients. Conversely, unilateral hypersecretion may be associated with primary unilateral adrenal hyperplasia undetectable on imaging[57]. In a recent series, one in three patients undergoing adrenalectomy for PA had a lateralized aldosterone hypersecretion without a unilateral adenoma[22].

## Differential diagnosis

Hypertension with hypokalemia and suppressed renin is known as mineralocorticoid hypertension, the mineralocorticoid involved being aldosterone in the vast majority of cases [58]. PA is easily excluded by the absence of aldosterone hypersecretion in cases of mineralocorticoid hypertension due to an excess secretion of cortisol or deoxycorticosterone[59], of which some can be corrected surgically; in apparent mineralocorticoid excess[60]; during exposure to glycyrrhizic acid (liquorice)[60]; or in pseudo-hypermineralocorticoidism due to Liddle syndrome [61].

## Management including treatment

### Treatment objectives

Treatment objectives in patients with PA are to reduce BP, correct hypokalemia, and to prevent or reverse the eventual cardiovascular or renal alterations caused by aldosterone excess. In retrospective case-control studies, the cardiovascular and renal consequences of hypertension were reported to be more severe in patients with PA than in patients with essential hypertension and similar levels of office BP [63,64]. Thus, correcting for aldosterone hypersecretion is a treatment objective *per se *[65]. Whether PA is associated with an increased prevalence of glucose metabolism disorders is still disputed [66].

In patients with lateralized aldosterone hypersecretion, this goal can be achieved by adrenalectomy and probably by the long-term prescription of aldosterone antagonists[67]. Patients' preferences should be taken into account. Candidates for surgery should be told that the presence of an aldosterone-producing adenoma poses no threat of cancer, that surgery may not cure their hypertension completely, and that the frequency of complications for laparoscopic adrenalectomy is about 8%.

### Adrenalectomy

#### Procedures

With regard to the risk of hypokalemia-induced arrhythmia during anesthesia, hypokalemic patients should be provided potassium chloride or aldosterone antagonists before surgery.

A complete unilateral adrenalectomy is required in patients with primary unilateral adrenal hyperplasia[68]. It is also preferable to adenoma enucleation in cases in which computed tomography has shown a Conn's adenoma, as multiple adenomas are frequent and are not necessarily identified by preoperative imaging[69]. Besides, the adrenal gland is a small organ, and devascularizing an adenoma frequently results in the devascularization of the entire gland, making conservative surgery difficult. Laparoscopic surgery, using transperitoneal [70] or retroperitoneal[71] approaches, is currently the procedure of choice. Mean operating time and length of hospitalization are typically 90 min and 4 days [22,70], respectively. The mean complication rate is 8% [20,70]. Complications of laparoscopic surgery include conversion to open surgery, hematoma due to intraoperative vascular injury, thromboembolism, pneumothorax or hemothorax, with most complications being benign.

#### Morphological/histological findings

The most common aspect is a unilateral, yellow, lipid-laden adenoma varying in diameter from 5 to 35 mm. Despite producing aldosterone, the tumor usually consists of zona fasciculata-type cells although zona glomerulosa- or mixed cell-type tumors have been described. Aldosterone-secreting adrenal carcinomas are extremely rare. These malignant tumors exceed 40 mm in size with involvement of local lymph nodes or invasion of adjacent organs [51].

#### Outcomes

Surgery abolishes aldosterone hypersecretion and hypokalemia in most patients with unilateral aldosterone hypersecretion [17,20-22,72]. It produces a large decrease in systolic BP (typically -20 to -40 mmHg), and in the number of antihypertensive medications prescribed (typically -1 therapeutic class) [19-22].

**Figure 5:**
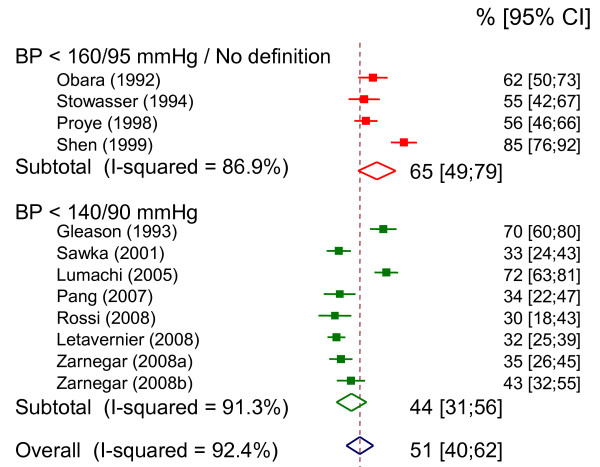
**Blood pressure outcome of adrenalectomy in primary aldosteronism**. Percentages of patients with hypertension cure following surgery in studies with more than 50 patients. Studies are classified according to the definition used for hypertension cure. One study was discarded[79] because of a large overlap with a more recent study[22]. One study involved patients from two centers as a derivation sample and a validation sample respectively [24]. Due to significant heterogeneity across studies, a random effects model was used to estimate the combined effects.

Patients should be warned that hypertension is not always cured. Of eleven studies involving 50 or more patients with PA followed up after unilateral adrenalectomy, seven correctly defined hypertension cure as BP <140/90 mmHg without treatment, and four used alternative definitions or did not report the criteria used to define normotension (Figure 5). The rate of hypertension cure was 51% [95%CI 40;62] overall, 44% [95%CI 31;56] in series using the standard definition of normal BP (<140/90 mmHg) [16,18-24,72] and 65% [95%CI 49;79] in the other series[15,17,73,74]. In cases in which an adrenalectomy does not cure hypertension, it usually leads to a clinically relevant improvement in the control of hypertension, with lower BP levels and/or less antihypertensive medication required. Seven of the 11 studies reported that BP improved without cure in 18 to 71% of patients, leading to hypertension benefit (cure or improvement) in 74 to 100% of operated patients.

#### Prediction of blood-pressure outcome

A dominant aldosterone-dependant component of hypertension and a low probability of associated essential hypertension are predictive of a better BP outcome after adrenalectomy.

In univariate analysis, several patient characteristics that suggest aldosterone-dependent hypertension have been associated with a favorable outcome: they include the presence of a typical and large aldosterone-producing adenoma on imaging studies or at pathological examination [15,17,20,24,72]; high urinary aldosterone excretion, low plasma renin or low serum potassium levels[18,22,72]; the absence of an increase in the plasma aldosterone concentration after standing up[74] and the preoperative normalization of BP on monotherapy with high-dose spironolactone[17,75]. Conversely, non-specific characteristics usually present in essential and/or severe hypertension have been associated with a poor BP outcome of adrenalectomy: they include older age [15,17-20,72,74-76] or longer duration of hypertension before surgery[17-19,21,22,72]; higher body-mass index [21,22,72] male sex [15,20,72,74]; a history of essential hypertension in first-degree relatives[17,18]; preoperative BP[17,21] and number of prescribed antihypertensive drugs [17-20,72]; and the presence of remodeling of resistance arteries[21].

Various factors have each been associated with a less favorable BP outcome in at least one multivariate model: lower urinary aldosterone excretion; a small adenoma or the presence of contralateral morphological abnormalities; the absence of BP control in patients on spironolactone; a higher number of antihypertensive medications required to control BP; older age or a longer history of hypertension; higher body mass index; male sex; and the presence of a family history of hypertension[15,17,18,20,22,72]. The validity of these multivariate analyses is threatened by small sample sizes; comparisons across studies are difficult because the various models did not include the same variables.

The relevance of these prognostic markers for selecting patients for surgery should not be overemphasized, as they do not take into account the invariable cure of hypokalemia and hyperaldosteronism by adrenalectomy and provide only a weak prediction of the BP benefit in individual patients. For example, according to the only prediction model validated to date, number of antihypertensive medication ≤ 2, a body mass index ≤ 25 kg/m^2^, a duration of hypertension ≤ 6 years and female sex are the best predictors of hypertension cure following adrenalectomy[24]. However, even if none of these features was present in an individual patient, this patient still had a 25% probability of being completely cured by an adrenalectomy and if not cured, hypertension was almost always better controlled. In another study in which the mean systolic BP decrease was -25 mmHg after surgery, the clinical impact of statistically significant prognostic factors was limited: the mean systolic BP decrease was only 3 mmHg less (-22 instead of -25 mmHg) in patients with a 0.5 mmol/l higher level of serum potassium before surgery, the most powerful predictor of unfavorable outcomes[22].

#### Long-term benefits

Operated patients can expect to be completely or partially weaned from mineralocorticoid antagonists or non-specific antihypertensive medication. The alternative to surgery is lifelong medication intended to correct or prevent the deleterious direct or indirect effects of hyperaldosteronism. Younger patients have a longer life expectancy and therefore derive a greater benefit from surgery. They also carry the smallest anesthetic risk. Early diagnosis of lateralized PA is therefore of paramount importance. The benefit-risk ratio is more balanced in older patients, especially if their antihypertensive medication has compelling indications, such as beta-blockers for coronary artery disease or angiotensin-converting enzyme and spironolactone for heart failure.

### Alternatives to adrenalectomy

Mineralocorticoid receptor antagonists - spironolactone and eplerenone - provide a specific treatment for PA in patients who are not candidates for surgery. Unfortunately, only a few of these patients show a good BP response to spironolactone monotherapy [77]. Furthermore, long-term tolerance of spironolactone at doses exceeding 50 mg per day is poor[78]. There is no published evidence to suggest that high doses of eplerenone are more effective and better tolerated than spironolactone in patients with PA. If necessary, lower doses of aldosterone receptor antagonists may be associated with non-specific antihypertensive agents.

## Unresolved questions

The etiopathogenesis and genomics of PA and aldosterone-producing adenomas are currently being studied by several research groups. Collaborative prospective studies are needed to document and standardize critical steps in the diagnosis of PA and the confirmation of lateralized PA in relation to surgically correctable PA. This specifically applies to studying the advantages that post-suppression plasma aldosterone concentrations have over basal concentrations, and the cut-off values that are used to detect a clinically relevant lateralizing ratio at AVS. As the rate of cure of hypertension following adrenalectomy is only 50%, there is also a need for randomized trials comparing the safety, acceptability and efficacy of surgery and aldosterone antagonists, regarding BP and target organ damage.

## Conclusions

Surgically correctable PA is sought in patients with hypokalemic or difficult-to-treat hypertension, and is diagnosed by the presence of unilateral aldosterone hypersecretion at AVS. Surgery is particularly useful for young PA patients, who can be completely cured, and for PA patients with resistant hypertension, whose BP control can be markedly improved.

## Competing interests

The authors declare that they have no competing interests.

## Authors' contributions

The authors equally contributed to this review article. They read and approved the final version of the manuscript.
